# Epidemiology, Patient Characteristics, Real-World Treatment Patterns, and Healthcare Utilization and Spending for Patients with Multifocal Motor Neuropathy: A US Claims-Based Analysis

**DOI:** 10.36469/001c.158137

**Published:** 2026-04-03

**Authors:** Nikhil Khandelwal, Caroline Geremakis, Faisal Riaz, Gina Ryan, Vishal Saundankar, Richard Sheer, Brandon Suehs

**Affiliations:** 1 Takeda Pharmaceuticals USA, Inc., Lexington, Massachusetts; 2 Takeda (United States); 3 Humana Healthcare Research Inc., Louisville, Kentucky

**Keywords:** costs, epidemiology, healthcare resource utilization, multifocal motor neuropathy, patient characteristics, real-world evidence, treatment

## Abstract

**Background:**

Multifocal motor neuropathy (MMN) is a rare, progressive neurological disease characterized by asymmetrical limb weakness. The real-world healthcare burden of MMN is not well established.

**Objectives:**

To characterize the epidemiology, diagnostic procedures, treatment patterns, healthcare resource utilization (HCRU), and healthcare spending associated with MMN in patients in the US.

**Methods:**

This retrospective, observational claims study extracted data from the Humana Healthcare Research Database, comprising US Medicare Advantage plan members. Eligible patients were aged 18-89 years, had ≥2 nondiagnostic medical claims (the first being the index date) associated with an MMN diagnosis code (January 1, 2017–June 30, 2022), and continuous enrollment for 12 months pre-index (baseline) and post-index (follow-up). Patients with amyotrophic lateral sclerosis, chronic inflammatory demyelinating neuropathy, or immunosuppressant use were excluded. Outcomes were assessed during the baseline and follow-up periods.

**Results:**

Deidentified data were extracted for 248 patients with MMN. Median (Q1, Q3) age at index was 70.0 (62.0, 77.0) years; most patients were male (53.6%) and White (78.2%). Diagnostic procedures included (baseline/follow-up periods) spinal magnetic resonance imaging (21.4%/18.1%), nerve conduction studies (19.8%/14.5%), and electromyography (17.7%/15.3%). Anticonvulsants, pain medications, corticosteroids, and central muscle relaxants were the most commonly used medications. Overall, 5.2% of patients had intravenous immunoglobulin (IVIG) during follow-up. Mean (standard deviation [SD]) time from index to IVIG initiation was 63.1 (52.2) days, with 6.5 (5.4) administrations, 28.7 (22.9) days between administrations, and 147.5 (133.9) days of total treatment. For all-cause HCRU, 23.8% of patients had ≥1 inpatient stay in the baseline period, with mean (SD) length of stay of 12.7 (14.5) days; during follow-up, 27.8% of patients had ≥1 inpatient stay (length of stay, 13.4 [16.2] days). During the baseline/follow-up periods, 43.1%/46.8% of patients had ≥1 emergency department visit, and 18.5%/28.6% used telehealth services. Median all-cause spending (baseline/follow-up) was 11 299/16 074 for total healthcare, 6745/10 630 for medical resources, and 1374/1701 for pharmacy.

**Discussion:**

Further studies are needed to enhance our understanding of the real-world diagnostic and treatment patterns associated with MMN and to determine long-term clinical outcomes.

**Conclusion:**

These real-world data highlighted the considerable burden associated with MMN on the healthcare system and patients.

## INTRODUCTION

Multifocal motor neuropathy (MMN) is a rare and progressive disease. Real-world data on the prevalence of MMN in the US are lacking, but studies from other countries have reported estimated prevalences of 0.3 to 0.4 cases per 100 000 in Japan,^1,2^ 0.6 per 100 000 in the Netherlands,^3^ and 1.3 per 100 000 in Australia.^4^ MMN is characterized by asymmetrical limb weakness, preferentially affecting the distal upper limbs.^5^ This leads to impaired fine motor skills in the hands of patients, which can affect many aspects of life.^6^

MMN is a chronic condition that often requires long-term treatment with intravenous immunoglobulin (IVIG) to manage symptoms and slow disease progression.^5,7,8^ Hence, early recognition and initiation of appropriate treatment are critical prognostic factors for patients.^3,7,9^ However, timely diagnosis remains challenging because the signs and symptoms of MMN overlap with those of other immunologic neuropathies, such as amyotrophic lateral sclerosis and chronic inflammatory demyelinating neuropathy.^5,10–12^ Misdiagnosis of MMN can have significant consequences for patients, including missed opportunities to prevent irreversible disability and exposure to treatments that may worsen symptoms.^8,12,13^ Moreover, although the hallmark electrophysiological finding of MMN is motor nerve conduction block at noncompressible sites, this may not be detectable in all patients.^5,10,12^ In these cases, additional procedures are required to support differential diagnosis, such as cerebrospinal fluid analysis, imaging studies, or MMN-associated antibody tests.^10^

The patient journey to obtaining an MMN diagnosis is often complex, involving multiple tests, treatments, and interactions with healthcare providers.^8^ However, the rarity of MMN means there is limited real-world evidence on the diagnostic and treatment patterns, healthcare resource utilization (HCRU), and healthcare spending for patients with the disease. The existing data have included patient populations outside the US, and these parameters have generally been investigated only in small cohort studies.^3,11,14,15^ Within the US, there is therefore a need to understand the real-world effect of MMN at both the patient and healthcare system levels.

In this study, we aimed to examine the clinical characteristics, diagnostic and treatment patterns, HCRU, and healthcare spending associated with MMN in patients enrolled in a US Medicare Advantage (MA) plan.

## METHODS

### Study Design and Population

**Figure 1.**
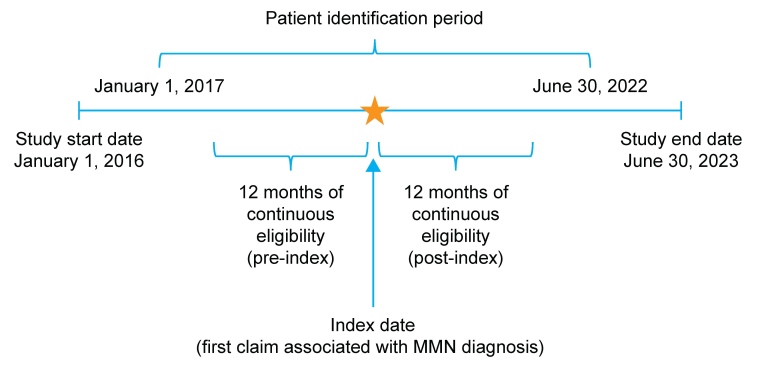
Study Design Abbreviation: MMN, multifocal motor neuropathy. Patients with any evidence of MMN during the baseline period were excluded.

This retrospective, observational study used administrative claims data obtained between January 1, 2016, and June 30, 2023, to identify individuals with newly diagnosed MMN (**Figure 1**). The Humana Healthcare Research Database, which includes information on enrollment, medical claims, and pharmacy claims for US MA plan members, was used for this study. Data were compliant with the Health Insurance Portability and Accountability Act (HIPAA). The study protocol was reviewed and approved by the Human Subject Protection Office, which follows HHS regulation 45 CFR §46 and the Office for Human Research Protections guidance on Coded Private Information or Specimens Use in Research (2008), before study initiation. No data were collected by direct contact with patients or their providers.

Patients were eligible for inclusion in the study if they were aged 18-89 years as of the index date, enrolled in an MA plan, and had ≥2 nondiagnostic medical claims >30 days apart that were associated with a diagnosis code for MMN (*International Classification of Diseases, Tenth Revision, Clinical Modification* [ICD-10-CM] code G61.82) between January 1, 2017, and June 30, 2022.

The Humana MA database includes data for Medicare Part C plans, which cover services provided by Medicare Part A (hospital insurance), Part B (medical insurance; buy-and-bill), and Part D (prescription drug). Thus, all of the submitted claims for IVIG infusion administered at provider offices and hospital outpatient departments were captured in medical claims using Healthcare Common Procedure Coding System (HCPCS) codes for IVIG treatments. If a patient received IVIG through a specialty pharmacy, such claims were identified using National Drug Code codes in pharmacy claims.

The index date was defined as the date of the first nondiagnostic medical claim associated with an MMN diagnosis. Nondiagnostic claims were defined as those billed for any medical services except for the administration of a diagnostic procedure or laboratory test (identified using Common Procedural Terminology [CPT], ICD-10 Procedure Coding System [PCS] and Logical Observation Identifiers Names and Codes [LOINC]; **Supplementary Table S1)**. This approach was taken to avoid inclusion of false MMN cases in this study. Monthly enrollment records, including details on health plan type, were available for all plan members. The follow-up period was defined as 12 months of continuous enrollment from the index date. If a patient died or disenrolled from the insurance plan, or if the study period ended before the completion of 12 months, data for that patient were excluded from the analysis. The 12-month pre-index and post-index periods are hereafter referred to as the baseline and follow-up periods, respectively. Patients were excluded from the study if they had ≥2 nondiagnostic claims associated with MMN during the baseline period, including the index date. Data for patients with ≥1 claim related to amyotrophic lateral sclerosis or with ≥2 claims related to chronic inflammatory demyelinating neuropathy during the baseline or follow-up periods were also excluded to minimize misclassification and ensure cohort specificity for MMN (**Supplementary Table S2**). The clinical presentation of MMN may mimic amyotrophic lateral sclerosis or chronic inflammatory demyelinating neuropathy; however, these diseases differ from MMN in prognosis and treatment.^16^ Data for patients with claims for immunosuppressant use or any diagnostic or procedural claims related to pregnancy during the baseline period were also excluded.

### Baseline Demographics and Clinical Characteristics

Patient demographics and clinical comorbidities were assessed on the index date using the baseline period. Age in years, sex, and geographic location were determined as of the index date. Population density was identified by matching the Rural-Urban Commuting Area Codes to the address ZIP Codes as of the index date. Dual eligibility status for Medicare and Medicaid and eligibility for the Low-Income Subsidy (LIS) were identified using data received from the Centers for Medicare and Medicaid Services.

The presence of comorbidities during the baseline period was assessed for each patient using the Deyo-Charlson Comorbidity Index (DCI) and Elixhauser Comorbidity Score, which were calculated based on inpatient and outpatient claims data. Indices were defined based on criteria by Charlson et al (DCI)^17^ and Elixhauser et al (Elixhauser Comorbidity Score).^18^ Composite scores, reflecting broader health risk, were also calculated for both indices, by combining their individual algorithms with methodology from Klabunde et al.^19^ Claims with appropriate diagnosis/procedure codes were used in the calculation of the DCI or Elixhauser scores if they met the following criteria: the code appeared on a claim with a place of treatment indicating inpatient hospitalization or the code appeared on ≥2 outpatient claims ≥30 days apart (with outpatient defined as any place of treatment that is not inpatient hospital). To protect the privacy of individuals, baseline demographics and clinical characteristics with counts <11 were masked.

Conditions that could be misdiagnosed as MMN, referred to here as MMN-mimic conditions, were also identified using ICD-10-CM codes associated with medical claims during the baseline and follow-up periods. A full list of conditions that were included is provided in **Supplementary Table S2**.

### Diagnostic and Treatment Patterns

Diagnostic procedures and treatments received were identified during the study. All diagnostic procedures were identified using CPT codes found on the medical claims during the follow-up period, and proportions of patients receiving each procedure or treatment were reported for the baseline and follow-up periods. Claims for MMN-specific antibody tests were identified using LOINC found on the laboratory claims.

The specialty of the provider submitting the index pharmacy or medical claim was determined based on the National Provider Identifier, which was categorized based on the HIPAA taxonomy code.

All treatments were identified from medical or pharmacy claims during the follow-up period using appropriate HCPCS/Generic Product Identifier/National Drug Code/CPT codes. Data for IVIG use were captured using HCPCS codes, including the number of IVIG administrations, the time between administrations, the duration of treatment, and the persistence to treatment. Time to initiation of IVIG treatment was measured from the index date. Treatment duration was defined as the number of days from the first administration to the last administration overall or before the start of a therapy gap of ≥60 days (90 days was used as a sensitivity measure). Therapy gaps were defined as a patient who received an administration but did not receive another in the following 60 (or 90) days. Persistence was defined as the number of days on IVIG treatment from treatment initiation to discontinuation or a therapy gap of >60 days.

The site of IVIG administration was identified using the place of service code associated with the pharmacy or medical claim. Other medications (eg, immunomodulators, oral corticosteroids, and plasma exchange) received during the baseline and follow-up periods were also assessed.

### Healthcare Resource Utilization

All-cause HCRU was assessed during the baseline and follow-up periods based on place of service, HCPCS/CPT, and revenue codes. Assessment of HCRU included inpatient stays, length of stay (LOS), physician office visits, emergency department visits, telehealth services, and other outpatient visits (comprising all visits other than physician outpatient appointments and telehealth services). LOS was calculated as the number of days between admission and discharge dates for inpatient admissions (calculated as the sum of hospital stays for all admissions during the measurement period) and for hospitalizations (calculated by dividing the total number of inpatient days by the total number of inpatient admissions during the measurement period).

Claims with an MMN diagnosis code in one of the first three positions were considered for the MMN-related HCRU analysis. MMN-related HCRU was measured during the 12-month follow-up period.

### Healthcare Spending

All-cause medical, pharmacy, and total healthcare spending were determined separately for the 12-month baseline and follow-up periods and adjusted to 2023 US dollars utilizing the Medical Consumer Price Index. All-cause medical claims encompassed all claims relating to inpatient, emergency department, and outpatient visits. MMN-related healthcare spending was defined as claims with a diagnosis code for MMN in one of the first three positions on the claim and was measured during the 12-month follow-up period.

### Statistical Analyses

This study included descriptive analyses of the study measures. For continuous study measures, mean (standard deviation [SD]) and median (Q1, Q3) were reported. Where appropriate, 95% confidence intervals (CIs) and minimum and maximum values were reported. For categorical variables, counts and proportions (percentages) were reported. Approximately one-third of patients in the full study cohort had a cancer diagnosis during either the baseline or follow-up period, which may have influenced HCRU and healthcare spending patterns; therefore, a sensitivity analysis was conducted for these outcomes after excluding those with a cancer diagnosis.

## RESULTS

### Baseline Demographic and Clinical Characteristics

Of 539 patients with ≥2 nondiagnostic medical claims for MMN, 248 (46.0%) met the eligibility criteria and were included in the study (**Supplementary Figure S1**). The median (Q1, Q3) age at study entry (index) was 70.0 (62.0, 77.0) years; 53.6% of patients were male, and most were White (78.2%). The majority of patients lived in the Southern US region (67.7%), and half resided in urban areas (50.8%) (**Table 1**). In total, 31.9% of patients were eligible for the Medicare LIS, and 25.4% were eligible for dual Medicare–Medicaid coverage. The mean (SD) baseline Elixhauser Comorbidity Score was 4.3 (3.2) (**Table 2**).

**Table 1.** Summary of Patient Demographics and Socioeconomic Characteristics in Patients with MMN

**Demographic/Characteristic**	**Overall MMN Cohort (N = 248)^a^**
Age, years	
Mean (SD)	68.9 (10.3)
Median (Q1, Q3)	70.0 (62.0-77.0)
Sex, n (%) [95% CI]	
Male	133 (53.6) [47.4-59.8]
Female	115 (46.4) [40.2-52.6]
Race, n (%) [95% CI]	
White	194 (78.2) [73.1-83.3]
Black	34 (13.7) [9.4-18.0]
Underrepresented race and ethnicity	NR
Missing	NR
Geographic region, n (%) [95% CI]	
South	168 (67.7) [61.9-73.5]
Midwest	46 (18.5) [13.7-23.3]
Northeast	NR
West	NR
Population density, n (%) [95% CI]	
Urban	126 (50.8) [44.6-57.0]
Suburban	72 (29.0) [23.4-34.6]
Rural	NR
Missing	NR
Low-Income Subsidy, n (%) [95% CI]	79 (31.9) [26.1-37.7]
Dual Medicare/Medicaid eligibility, n (%) [95% CI]	63 (25.4) [20.0-30.8]

Abbreviations: CI, confidence interval; MMN, multifocal motor neuropathy; NR, not reported; SD, standard deviation.

NR indicates values masked due to small counts.

^a^To protect the privacy of individuals, counts <11 have been masked; values cannot be deduced from those in other rows.

**Table 2.** Patient Comorbidities During Baseline Period in Patients with MMN

**Characteristic**	**Baseline (N = 248)^a^**
Elixhauser Comorbidity Score, mean (SD) [95% CI]	4.3 (3.2) [3.9-4.7]
Elixhauser comorbidities, n (%) [95% CI]	
Hypertension (uncomplicated)	175 (70.6) [64.9-76.3]
Chronic obstructive pulmonary disease	71 (28.6) [23.0-34.2]
Diabetes (uncomplicated)	71 (28.6) [23.0-34.2]
Diabetes (complicated)	66 (26.6) [21.1-32.1]
Depression	63 (25.4) [20.0-30.8]
Peripheral vascular disease	59 (23.8) [18.5-29.1]
Obesity	56 (22.6) [17.4-27.8]
Cardiac arrhythmia	49 (19.8) [14.8-24.8]
Hypertension (complicated)	49 (19.8) [14.8-24.8]
Renal failure	49 (19.8) [14.8-24.8]
Hypothyroidism	44 (17.7) [12.9-22.5]
Other neurological disorders	39 (15.7) [11.2-20.2]
Fluid electrolyte disorders	37 (14.9) [10.5-19.3]
Congestive heart failure	32 (12.9) [8.7-17.1]
Solid tumor (no metastasis)	32 (12.9) [8.7-17.1]
Drug abuse	23 (9.3) [5.7-12.9]
Valvular disease	20 (8.1) [4.7-11.5]
Deficiency anemia	19 (7.7) [4.4-11.0]
Rheumatoid arthritis, collagen disease	15 (6.0) [3.0-9.0]
Liver failure	14 (5.6) [2.7-8.5]
Pulmonary circulatory disorder	12 (4.8) [2.1-7.5]
Coagulopathy	11 (4.4) [1.8-7.0]
Alcohol abuse	NR
Paralysis	NR
Weight loss	NR
HIV/AIDS	NR
Blood loss anemia	NR
Lymphoma	NR
Metastatic cancer	NR
Peptic ulcer disease (no bleed)	NR
Psychoses	NR
DCI score, mean (SD) [95% CI]	2.5 (2.4) [2.2-2.8]
DCI comorbidities, n (%) [95% CI]	
Chronic obstructive pulmonary disease	71 (28.6) [23.0-34.2]
Peripheral vascular disease	59 (23.8) [18.5-29.1]
Diabetes with complications	57 (23.0) [17.8-28.2]
Renal disease	49 (19.8) [14.8-24.8]
Cancer (including leukemia and lymphoma)	32 (12.9) [8.7-17.1]
Cerebrovascular disease	32 (12.9) [8.7-17.1]
Congestive heart failure	32 (12.9) [8.7-17.1]
Diabetes without complications	22 (8.9) [5.4-12.4]
Dementia	14 (5.6) [2.7-8.5]
Myocardial infarction	13 (5.2) [2.4-8.0]
Mild liver disease	12 (4.8) [2.1-7.5]
Connective tissue disease	11 (4.4) [1.8-7.0]
Paraplegia and hemiplegia	NR
Metastatic carcinoma	NR
Moderate or severe liver disease	NR
Peptic ulcer disease	NR

Abbreviations: CI, confidence interval; DCI, Deyo-Charlson Comorbidity Index; MMN, multifocal motor neuropathy; NR, not reported; SD, standard deviation.

NR indicates values masked due to small counts.

^a^To protect the privacy of individuals, counts <11 have been masked; values cannot be deduced from those in other rows.

The most common Elixhauser comorbidities during the baseline period were hypertension without complications (70.6%), chronic obstructive pulmonary disease (28.6%), diabetes without complications (28.6%), diabetes with complications (26.6%), and depression (25.4%). In the baseline period, 75.8% of patients had ≥1 MMN-mimic condition, and the most common were ill-defined neuromuscular complaints (48.8%), radiculopathy (46.0%), unspecified polyneuropathy (35.5%), and sensory dysfunction (15.7%) (**Table 3**).

**Table 3.** MMN-Mimic Conditions During Baseline Period in Patients with MMN

**Characteristic**	**Baseline (N = 248)**
Any MMN-mimic differential diagnoses, n (%) [95% CI]	188 (75.8) [70.5-81.1]
Neurological conditions, n (%) [95% CI]	
Ill-defined neuromuscular complaint	121 (48.8) [42.6-55.0]
Radiculopathy	114 (46.0) [39.8-52.2]
Exclusion criteria for MMN diagnosis	97 (39.1) [33.0-45.2]
Polyneuropathy (unspecified)	88 (35.5) [29.5-41.5]
Hereditary and idiopathic neuropathy	39 (15.7) [11.2-20.2]
Diabetes mellitus with diabetic neuropathy	39 (15.7) [11.2-20.2]
Upper extremity neuropathies	19 (7.7) [4.4-11.0]
Other neuropathy	17 (6.9) [3.7-10.1]
Spinal stenosis	12 (4.8) [2.1-7.5]

Abbreviations: CI, confidence interval; MMN, multifocal motor neuropathy.

### Diagnostic and Treatment Patterns

The provider submitting the index claim related to an MMN diagnosis was a primary care provider in 59.7% of cases and a neurologist in 5.6% of cases (**Supplementary Table S3**). Claims associated with any of the MMN-related diagnostic procedures were submitted for fewer than a quarter of all patients, including magnetic resonance imaging (MRI), 23.0%; electrophysiological analysis, 21.4%; and MMN-associated antibody tests, 5.6%. The most common diagnostic procedures (baseline/follow-up) were spinal MRIs (21.4%/18.1%), nerve conduction studies (19.8%/14.5%), and electromyography (17.7%/15.3%).

Based on pharmacy claims, the most commonly received medications during the baseline period were anticonvulsants (58.1%), pain medications (56.0%), corticosteroids (29.8%), and central muscle relaxants (23.4%). The most commonly used medications were similar during the follow-up period, with most pharmacy claims being for anticonvulsants (65.3%), pain medications (64.9%), corticosteroids (37.1%), and central muscle relaxants (29.4%). Overall, 5.2% of patients with MMN received IVIG during the follow-up period. The mean (SD) time from index diagnosis of MMN to first IVIG prescription claim was 63.1 (52.2) days. IVIG treatment involved a mean (SD) of 6.5 (5.4) administrations, 28.7 (22.9) days between administrations, and a total treatment duration of 147.5 (133.9) days.

### All-Cause and MMN-Related Healthcare Resource Utilization

In the baseline period, 23.8% of patients had ≥1 all-cause inpatient stay (**Table 4**). The mean (SD) LOS was 12.7 (14.5) days per patient with an inpatient stay and 8.6 (11.0) days per hospitalization. In the follow-up period, 27.8% of patients had ≥1 inpatient stay. The mean (SD) LOS was 13.4 (16.2) days per patient with an inpatient stay and 8.5 (10.1) days per hospitalization. Overall, 97.2% (baseline) and 98.8% (follow-up) of patients had ≥1physician office visit, and, in both the baseline and follow-up periods, 95.2% had ≥1 outpatient visit. In total, 43.1% (baseline) and 46.8% (follow-up) of patients had ≥1 emergency department visit, and 18.5% (baseline) and 28.6% (follow-up) used telehealth services.

**Table 4.** All-Cause HCRU During Baseline and Follow-up Periods in Patients with MMN for Overall Population

**All-cause HCRU**	**Baseline (N = 248)**	**Follow-up (N = 248)**
Inpatient stay, n (%) [95% CI]	59 (23.8) [18.5-29.1]	69 (27.8) [22.2-33.4]
LOS in days, mean (SD) [95% CI]		
Total LOS during baseline period, all patients	3.0 (8.9) [1.9-4.1]	3.7 (10.4) [2.4-5.0]
Total LOS during baseline period, patients with inpatient stays	12.7 (14.5) [9.0-16.4]	13.4 (16.2) [9.6-17.2]
LOS per hospitalization	8.6 (11.0) [5.8-11.4]	8.5 (10.1) [6.1-10.9]
Physician office visit, n (%) [95% CI]	241 (97.2) [95.1-99.3]	245 (98.8) [97.4-100.2]
Outpatient visit,^a^ n (%) [95% CI]	236 (95.2) [92.5-97.9]	236 (95.2) [92.5-97.9]
Emergency department visit, n (%) [95% CI]	107 (43.1) [36.9-49.3]	116 (46.8) [40.6-53.0]
Telehealth services, n (%) [95% CI]	46 (18.5) [13.7-23.3]	71 (28.6) [23.0-34.2]

Abbreviations: CI, confidence interval; HCRU, healthcare resource utilization; LOS, length of stay; MMN, multifocal motor neuropathy; SD, standard deviation.

^a^Includes all outpatient visits other than physician outpatient appointments and telehealth services.

In the follow-up period, 55.4% (95% CI, 48.1-62.7) of patients had an MMN-related physician office visit, and 6.8% (95% CI, 3.1-10.5) used MMN-related telehealth services. MMN-related hospitalizations and emergency department visits were too infrequent in the follow-up period to support valid analyses.

### All-Cause and MMN-Related Healthcare Spending

**Figure 2.**
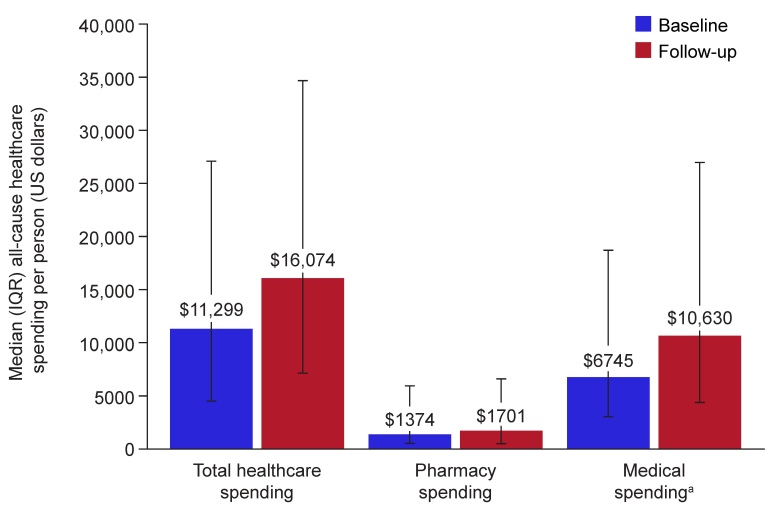
Median (IQR) All-Cause Healthcare Spending During Baseline and Follow-up Periods in Patients with MMN for Overall Population Abbreviations: IQR, interquartile range; MMN, multifocal motor neuropathy. ^a^Medical claims encompass all claims relating to inpatient, emergency department, and outpatient spending.

Median baseline/follow-up all-cause healthcare spending per person was $11 299/$16 074 for total healthcare, $6745/$10 630 for medical, and $1374/$1701 for pharmacy utilization (**Figure 2**). Outpatient spending (median) constituted most of the all-cause medical spending during baseline ($3561) and follow-up ($4664) periods. MMN-related healthcare spending in the follow-up period (median [Q1, Q3]) per person was $265 ($84, $713) for total healthcare, $241 ($0, $596) for medical, and $0 ($0, $0) for pharmacy utilization (MMN treatments). Mean all-cause healthcare spending is shown in **Supplementary Table S4**.

### Sensitivity Analyses

Overall, 28.6% of patients from the study sample (71/248) had a cancer diagnosis in either the baseline or follow-up periods. In sensitivity analyses that excluded these patients, patterns of HCRU and healthcare spending were generally similar in those without cancer (71.4% [n/N=177/248]; **Supplementary Table S4**) and the full study cohort (**Table 4** and **Figure 2**).

## DISCUSSION

To date, studies on treatment patterns, HCRU, and healthcare spending among patients with MMN have included patient populations outside the US. Due to the rarity of MMN, only a few small cohort (≤88 patients) studies have been able to investigate these parameters.^3,11,14,15^ To address this lack of real-world evidence among US patients, our study present data from a cohort of 248 patients with MMN enrolled in a US MA plan. Specifically, the data suggest that a substantial disease burden is experienced by patients with MMN during their diagnostic journey, as well as the associated effect of MMN on HCRU and healthcare spending.

Diagnosis of MMN is challenging and often delayed, with reported periods of over a year between the occurrence of first symptoms and a confirmed diagnosis.^11,12,14^ A major contributing factor to these delays may be related to the lack of differential diagnoses (ie, difficulty in distinguishing MMN from other conditions with similar signs and symptoms).^5,10–12^ Indeed, in our analysis, most (~76%) patients with MMN had claims for MMN-mimic neurological conditions in the baseline period, which may further highlight the ongoing need to raise awareness of how to distinguish MMN from other symptomatically similar conditions.

The majority (59.7%) of index diagnoses came from primary care providers, and only a small (5.6%) proportion of patients had index diagnoses from neurologists. Only 19.8% of patients underwent a nerve conduction study in the baseline period, and use of other diagnostic tests was similarly low (eg, spinal MRI, 21.4%; electromyography, 17.7%). This highlights the need for more appropriate referrals to specialist centers for MMN diagnosis in the Humana MA plan population.^7,20,21^ It may also indicate a need for better awareness among diagnosing physicians of the current European Federation of Neurological Societies/Peripheral Nerve Society (EFNS/PNS) guidelines for MMN (last updated in 2010), which consider both clinical examination and electrophysiological tests as key diagnostic tests in patients with suspected MMN.^8^ With this in mind, it is critical that healthcare professionals who are likely to see patients with suspected MMN receive clear information on the importance of referral and early diagnosis, to prevent exacerbation of irreversible disability and to reduce the burden on patients and the healthcare system.^12,13^

Nerve conduction tests and other diagnostic procedures were rarely performed in the follow-up period, consistent with previous studies.^11,15,22^ This may further suggest an area for improvement, given the usefulness of such procedures in monitoring disease progression and guiding treatment decisions.^7,21^ Strikingly, in the present study, only 5.2% of patients with MMN received IVIG following their diagnosis. We appreciate that claims data cannot provide information on disease severity or the appropriateness of clinical decision-making. However, if these observations are reflective of real-world practice, it would be in contrast to a good practice point in the EFNS/PNS 2010 guidelines, which proposes the use of IVIG as first-line therapy in this patient population.^8^ Indeed, a retrospective US claims analysis of the Optum Research Database, published in 2025, showed that 16.4% of patients with MMN (n/N = 55/336) received IVIG therapy in the year before their diagnosis, and 28.0% (n/N = 94/336) received IVIG in the year after the diagnosis.^23^ A possible explanation for the low use of IVIG in the present analysis is limited access to treatment due to financial/economic barriers. Nearly one-third of patients qualified for LIS, and a quarter of patients were dually eligible for Medicare and Medicaid, indicators of financial hardship that may have restricted access to care. Consequently, the observed patterns of treatment initiation, persistence, and discontinuation may also reflect structural and social determinants as well as intrinsic treatment effects. The low rates of IVIG use may also reflect the higher levels of MMN diagnosis/management by primary care physicians than by neurologists. In addition, the use of IVIG was low in our study population (newly diagnosed patients). This may be because the degree of disability in these individuals was not considered severe enough to warrant IVIG treatment.^8^ The use of medications such as anticonvulsants, painkillers, and corticosteroids (but not central muscle relaxants) was more common in the follow-up period than the baseline period, and notably, approximately one-third of patients received corticosteroids in the study period (baseline, 29.8%; follow-up, 37.1%). Although studies have shown no benefit and even clinical worsening with corticosteroid treatment in patients with MMN,^7,24^ their use in this context may have been for the management of one of the neurological or non-neurological comorbidities observed in this study cohort.

US patients with MMN in our study had a substantial number of all-cause inpatient hospitalizations and emergency department visits before and after diagnosis, which were key drivers of HCRU and healthcare spending. A study from Mahdi-Rogers et al has previously demonstrated significant HCRU and healthcare spending in a 2008 UK study population.^25^ The majority of healthcare interactions in our analysis (for the 12-month baseline and follow-up periods) were outpatient visits (baseline/follow-up, 95.2%/95.2%), rather than inpatient visits (baseline/follow-up, 23.8%/27.8%). This is consistent with data from a UK study conducted over a 6-month period (outpatient visits, 77.8%; inpatient visits, 44.4%). For individuals with MMN who received IVIG in the UK study (44.4% of the patient population), annual total spending per patient was reported as £49 430 (equivalent to $91 584, based on the 2008 UK sterling to US dollar exchange rate provided by the UK Office for National Statistics).^25,26^ Although the median total healthcare spending in the Humana MA patient population was $16 074, only 5.2% of patients received IVIG. Therefore, the total healthcare spending cost reported in our analysis may be more comparable to the patient population that did not receive IVIG from the UK-based study (£9046 [2008 US dollar conversion: $14 501]).^25,26^ As MMN is a chronic condition that often requires long-term treatment with IVIG to manage symptoms and disease progression,^5,7,8^ these data should be interpreted with caution. This is owing to the differences between studies in currency and their relative value over time, methodological approach (including study duration), and the underlying patient populations.

Limitations of this analysis include those inherent to analyses of claims data, such as the potential lack of generalizability of treatment and HCRU patterns of Humana MA members to other settings, such as commercial populations, younger populations, or those who are uninsured. It should also be noted that the geographical spread of patients was not fully representative, with very few patients from the Northeast or West.

This analysis included patients who had ≥2 MMN claims. A similar approach was used in a prior analysis of MMN conducted in a different US-based administrative claims study.^23^ Laboratory results and nerve conduction findings that would be needed for a systematic validation of the diagnostic code for MMN were not available in this claims database. We considered conducting a sensitivity analysis using a sample of ≥3 MMN claims to assess potential misclassification risk. However, initial analyses suggested that this would be unlikely to have affected the results, would have halved the sample size, and resulted in several study measures being masked due to low numbers and privacy concerns.

In addition, a non-MMN-matched comparator cohort (eg, similar age/comorbidity profile without MMN) would have provided valuable context for understanding the healthcare utilization and cost burden. Unfortunately, such comparator analyses would have significantly reduced the sample size during matching.

Data on MMN-related HCRU in the follow-up period were limited; hence, it should be acknowledged that the all-cause HCRU may reflect (at least in part) the impacts of comorbidities. In addition, our study may be affected by survivor bias, owing to the need for continuous enrollment during the 12-month baseline and follow-up periods. Patients with a history of cancer were not excluded from the main analysis of this study, which may have influenced HCRU and healthcare spending patterns. However, results of a sensitivity analysis excluding patients with a cancer diagnosis suggested that HCRU and healthcare spending patterns were generally similar in patients without cancer and the full study cohort. The 12-month follow-up period may have underestimated both IVIG use and long-term resource utilization. A future study looking at a longer follow-up period may be of interest.

## CONCLUSION

Overall, real-world data from this study have highlighted the considerable burden on the healthcare system and on individual patients with MMN. Given the importance of early diagnosis and effective treatment as long-term prognostic factors for patients with MMN,^3,7,9^ there is a need to raise awareness within primary care settings. This includes highlighting the value of early referral to specialist care centers for those with suspected MMN, recognizing comorbidities associated with the disease, and understanding appropriate treatment options. Additional real-world studies are required to improve our understanding of diagnostic and treatment patterns in patients with MMN in general and to determine long-term clinical outcomes and their implications for patients and the healthcare system.

### Disclosures

N.K., C.G., F.R., and G.R. are employees of Takeda Pharmaceuticals USA, Inc., Lexington, Massachusetts, USA, and are stockholders of Takeda Pharmaceutical Company Limited. V.S. and R.S. are employees of Humana Healthcare Research Inc., Louisville, Kentucky, USA. B.T.S. is an employee of Humana Healthcare Research Inc., Louisville, Kentucky, USA, and is a Humana shareholder.

### Ethical Approval

This study was conducted in accordance with ethical principles that have their origin in the Declaration of Helsinki, Good Clinical Practice (GCP), the International Society for Pharmacoepidemiology (ISPE) Guidelines for Good Pharmacoepidemiology Practice (GPP), and any applicable local regulations. The Humana Human Subject Protection Office determined that this research did not constitute human subjects research in compliance with applicable laws, ethical standards, and Humana policies; therefore, it was considered exempt from institutional review board review.

### Reporting Guidelines

This study followed the Strengthening the Reporting of Observational Studies in Epidemiology (STROBE) guidelines for reporting observational studies.

## Supplementary Material

Online Supplementary Material

## Data Availability

Takeda does not plan to share data supporting the results reported in this article.

## References

[1] Matsui N. (2012). [Multifocal motor neuropathy: current review of epidemiology and treatment]. Rinsho Shinkeigaku.

[2] Aotsuka Y., Misawa S., Suichi T.. (2024). Multifocal motor neuropathy in Japan: a nationwide survey on prevalence, clinical profiles, and treatment. Muscle Nerve.

[3] Cats E. A., van der Pol W. L., Piepers S.. (2010). Correlates of outcome and response to IVIg in 88 patients with multifocal motor neuropathy. Neurology.

[4] Park S. B., Li T., Kiernan M. C.. (2022). Prevalence of chronic inflammatory demyelinating polyneuropathy and multifocal motor neuropathy in two regions of Australia. Muscle Nerve.

[5] Allen J. A., Clarke A. E., Harbo T. (2024). A practical guide to identify patients with multifocal motor neuropathy, a treatable immune-mediated neuropathy. Mayo Clin Proc Innov Qual Outcomes.

[6] Wonink H. A., Kruithof W. J., Goedee H. S., Beelen A. (2023). Chronic inflammatory neuropathies and their impact on activities and participation. Eur J Neurol.

[7] Yeh W. Z., Dyck P. J., van den Berg L. H., Kiernan M. C., Taylor B. V. (2020). Multifocal motor neuropathy: controversies and priorities. J Neurol Neurosurg Psychiatry.

[8] Joint Task Force of the EFNS and the PNS (2010). European Federation of Neurological Societies/Peripheral Nerve Society guideline on management of multifocal motor neuropathy. Report of a joint task force of the European Federation of Neurological Societies and the Peripheral Nerve Society—first revision. J Peripher Nerv Syst.

[9] Van Asseldonk J. T., Van den Berg L. H., Kalmijn S.. (2006). Axon loss is an important determinant of weakness in multifocal motor neuropathy. J Neurol Neurosurg Psychiatry.

[10] Allen J. A., Parry G. J. (2015). Acquired immunologic neuropathies. Semin Neurol.

[11] Taylor B. V., Wright R. A., Harper C. M., Dyck P. J. (2000). Natural history of 46 patients with multifocal motor neuropathy with conduction block. Muscle Nerve.

[12] Lawson V., Robbins N. M. (2018). The potential misdiagnosis of multifocal motor neuropathy as amyotrophic lateral sclerosis–a case series. US Neurol.

[13] Lawson V. H., Arnold W. D. (2014). Multifocal motor neuropathy: a review of pathogenesis, diagnosis, and treatment. Neuropsychiatr Dis Treat.

[14] Stangel M., Gold R., Pittrow D.. (2016). Treatment of patients with multifocal motor neuropathy with immunoglobulins in clinical practice: the SIGNS registry. Ther Adv Neurol Disord.

[15] Slee M., Selvan A., Donaghy M. (2007). Multifocal motor neuropathy: the diagnostic spectrum and response to treatment. Neurology.

[16] Vlam L., van der Pol W. L., Cats E. A.. (2011). Multifocal motor neuropathy: diagnosis, pathogenesis and treatment strategies. Nat Rev Neurol.

[17] Charlson M. E., Pompei P., Ales K. L., MacKenzie C. R. (1987). A new method of classifying prognostic comorbidity in longitudinal studies: development and validation. J Chronic Dis.

[18] Elixhauser A., Steiner C., Harris D. R., Coffey R. M. (1998). Comorbidity measures for use with administrative data. Med Care.

[19] Klabunde C. N., Potosky A. L., Legler J. M., Warren J. L. (2000). Development of a comorbidity index using physician claims data. J Clin Epidemiol.

[20] Dyck P. J., Taylor B. V., Davies J. L.. (2015). Office immunotherapy in chronic inflammatory demyelinating polyneuropathy and multifocal motor neuropathy. Muscle Nerve.

[21] Beadon K., Guimaraes-Costa R., Leger J. M. (2018). Multifocal motor neuropathy. Curr Opin Neurol.

[22] Philibert M., Grapperon A. M., Delmont E., Attarian S. (2017). Monitoring the short-term effect of intravenous immunoglobulins in multifocal motor neuropathy using motor unit number index. Clin Neurophysiol.

[23] Khandelwal N., Sanchirico M., Ajibade A.. (2025). Characteristics, treatment patterns, healthcare resource utilization, and costs among patients with multifocal motor neuropathy: a US claims database cohort study. J Health Econ Outcomes Res.

[24] Azulay J. P., Rihet P., Pouget J.. (1997). Long term follow up of multifocal motor neuropathy with conduction block under treatment. J Neurol Neurosurg Psychiatry.

[25] Mahdi-Rogers M., McCrone P., Hughes R. A. (2014). Economic costs and quality of life in chronic inflammatory neuropathies in southeast England. Eur J Neurol.

[26] Office for National Statistics (2025). Average Sterling exchange rate: US Dollar XUMAUSS.

