# Supplementary material for: Epidemiology, Patient Characteristics, Real-World Treatment Patterns, and Healthcare Utilization and Spending for Patients with Multifocal Motor Neuropathy: A US Claims-Based Analysis
Source: J Health Econ Outcomes Res. 2026 Apr 3;13(1):111–9. doi: 10.36469/001c.158137 (PMC13050543; doi:10.36469/001c.158137)
Supplement: Online Supplementary Material [file jheor_2026_13_1_158137_338474.pdf]

Journal of Health Economics and Outcomes Research

Online Supplementary Material

Epidemiology, Patient Characteristics, Real-World Treatment Patterns, and Healthcare Utilization and Spending for Patients with Multifocal Motor Neuropathy: A US Claims-Based Analysis. *JHEOR*. 2026;13(1):111-119. doi:10.36469/jheor.2026.158137

Table S1: CPT, ICD-10-PCS, and LOINC Codes Used to Identify MMN Procedures and Lab Tests

Table S2: ICD-10-CM Codes for MMN-Mimic Conditions

Table S3: Diagnostic Patterns During Baseline and Follow-up Periods in Patients with MMN

Table S4: Mean Healthcare Spending During Baseline and Follow-up Periods for Patients with MMN for Overall Population

Table S5: All-Cause HCRU and Healthcare Spending During Baseline and Follow-up Periods for Patients with MMN and No Cancer Diagnosis

This supplementary material has been provided by the authors to give readers additional information about their work.

This is an open-access article distributed under the terms of the Creative Commons Attribution 4.0 International License (CCBY-4.0). View this license's legal deed at http://creativecommons.org/licenses/by/4.0 and legal code at http://creativecommons.org/licenses/by/4.0/legalcode for more information.

Table S1. CPT, ICD-10-PCS, and LOINC Codes Used to Identify MMN Procedures and Lab Tests

Procedure	CPT Code	ICD-10-PCS	LOINC
Electrophysiological procedures			
Electromyography	92265, 95860, 95861, 95863, 95864, 95867, 95868, 95869, 95870, 95872, 95885, 95886, 95887, 96002, 96003	R94131	
Nerve conduction studies	95905, 95907, 95908, 95909, 95910, 95911, 95912, 95913, 95933, 95937	G0255	
Nerve ultrasound	76883		
Nonspecific ultrasound	76882, 76970, 76999	BH47ZZZ, BH4CZZZ, BL40ZZZ, BP48ZZZ, BP49ZZZ, BP4GZZZ, BP4HZZZ, BP4LZZZ, BP4MZZZ, BP4NZZZ, BP4PZZZ, BR40ZZZ, BW4FZZZ	
Magnetic resonance imaging			
Spinal	72141, 72142, 72146, 72147, 72148, 72149, 72156, 72157, 72158, 72159	BP3MZZZ, BR30Y0Z, BR30YZZ, BR30ZZZ, BR31Y0Z, BR31YZZ, BR31ZZZ	
Other	73218, 73219, 73220, 73718, 73719, 73720	BL30Y0Z, BL30YZZ, BL30ZZZ, BP38Y0Z, BP38YZZ, BP38ZZZ, BP39Y0Z, BP39YZZ, BP39ZZZ, BP3EY0Z, BP3EYZZ, BP3EZZZ, BP3FY0Z, BP3FYZZ, BP3FZZZ, BP3GY0Z, BP3GYZZ, BP3GZZZ, BP3HY0Z, BP3HYZZ, BP3HZZZ, BP3JY0Z, BP3JYZZ, BP3JZZZ, BP3KY0Z, BP3KYZZ, BP3KZZZ, BP3LY0Z, BP3LYZZ, BP3LZZZ, BP3MY0Z, BP3MYZZ, BP3MZZZ	
Lumbar puncture–cerebrospinal fluid analysis	62270, 62328, 70015		
MMN-associated antibody tests			
Blood test for monoclonal gammopathies/GM1 antibodies	83520		21080-7, 31225-6, 43861-4, 10359-8, 31227-2, 30200-0, 13663-0, 31226-4, 44738-3, 48660-5, 29960-2, 43241-9, 51703-7, 63247-1, 89518-5, 29959-4, 43240-1, 51729-2, 63243-0, 89521-9, 50770-7, 63244-8, 89519-3, 51729-2, 51703-7, 43240-1, 88731-5, 6925-2, 88723-2, 31500-2
Biopsy			
Nerve biopsy	64795		

Abbreviations: CPT, Common Procedural Terminology; GM1, ganglioside; LOINC, Logical Observation Identifiers Names and Codes.

Table S2. ICD-10-CM Codes for MMN-Mimic Conditions

Condition	Code
Amyotrophic lateral sclerosis	G12.21x
Brachial plexus disorder	G54.0x
Carpal tunnel syndrome	G56.00x, G56.01x, G56.02x, G56.03x
Cervical root disorder	G54.d2x
Chronic inflammatory demyelinating polyneuritis	G61.81x
Diabetic neuropathy (unspecified)	E08.40x, E09.40x, E10.40x, E11.40x, E13.40x
Diabetic polyneuropathy	E08.42x, E09.42x, E10.42x, E13.42x
Drug-/substance-related neurotoxicity (drugs and other)	G620, G621, G622, G6282, G652
Drug-/substance-related neurotoxicity (lead)	R7871, T560X1A, T560X1D, T560X1S, T560X2A, T560X2D, T560X2S, T560X3A, T560X3D, T560X3S, T560X4A, T560X4D, T560X4S, Z77011
Guillain-Barré syndrome	G61.0x
Hereditary and idiopathic neuropathy (unspecified)	G60.9x
Hereditary motor and sensory neuropathy	G600
Hirayama disease	G128, G718, G719
Ill-defined neuromuscular complaint	R250, R251, R252, R253, R258, R259, R260, R261, R262, R2681, R2689, R269, R270, R278, R279, R290, R291, R292, R293, R294, R295, R296, R29700, R29701, R29702, R29703, R29704, R29705, R29706, R29706, R29707, R29708, R29709, R29710, R29711, R29712, R29713, R29714, R29715, R29716, R29717, R29718, R29719, R29720, R29721, R29722, R29723, R29724, R29725, R29726, R29727, R29728, R29729, R29730, R29731, R29732, R29733, R29734, R29735, R29736, R29737, R29738, R29739, R29740, R29741, R29742, R29810, R29818, R29898, R2990, R2991, R9089, R936, R937, R94130, R94131, R94138, R531
Inflammatory polyneuropathy (unspecified)	G61.89x, G61.9x
Lumbosacral plexus disorder	G54.1x
Lumbosacral root disorder	G54.4x
Median nerve disorder	G56.10x, G56.11x, G56.12x, G56.13x
Monoplegia	G83.1x, G83.2x, G83.3x
Motor neuron disease (unspecified)	G12.20
Nerve injury	S44.x
Neuropathy	G54.5x, G54.6x, G54.7x, G54.8x, G54.9x, G55.x
Other diabetic neuropathy	E0841, E0843, E0941, E0943, E1041, E1043, E1141, E1142, E1143, E1341, E1343
Other hereditary and idiopathic neuropathy	G60.3x, G60.8x
Other neuropathy	A5043, A5215, B0223, B2684, B2701, B2711, B2781, B2791, G602, G6281, G651, G9009, G990, H462, M0550, M05511, M05512, M05519, M05521, M05522, M05529, M05531, M05532, M05539, M05541, M05542, M05549, M05551, M05552, M05559, M05561, M05562, M05569, M05571, M05572, M05579, M0559, M3483
Polyneuropathy (unspecified)	G62.9x, G63.x
Porphyria	E800, E801, E8020, E8021, E8029
Progressive muscular atrophy	G12.25x
Radial nerve disorder	G56.30x, G56.31x, G56.32x, G56.33x
Radiculopathy (specified cause)	M4720, M4721, M4722, M4723, M4724, M4725, M4726, M4727, M4728, M47811, M47812, M47813, M47814, M47815, M47816, M47817, M47818, M47819, M48062, M5010, M5011, M5012, M50121, M50122, M50123, M5013, M5114, M5115, M5116, M5117
Radiculopathy (unlisted cause)	G580, M5410, M5411, M5412, M5413, M5414, M5415, M5416, M5417, M5418, M5430, M5431, M5432, M5440, M5441, M5442, M542
Sarcoid neuropathy or sarcoidosis (unspecified)	D8682, D8689, D869
Sarcoidosis (other site)	D860, D861, D862, D863, D8681, D8683, D8684, D8685, D8686, D8687

Table S2. ICD-10-CM Codes for MMN-Mimic Conditions

Condition	Code
Spinal stenosis	M48062
Ulnar nerve lesion	G56.20x, G56.21x, G56.22x, G56.23x
Upper limb causalgia	G56.40x, G56.41x, G56.42x, G56.43x
Upper limb mononeuropathy	G56.80x, G56.81x, G56.82x, G56.83x, G56.90x, G56.91, G56.92x, G56.93x

Abbreviation: ICD-10-CM, *International Classification of Diseases, Tenth Revision, Clinical Modification*.

Table S3. Diagnostic Patterns During Baseline and Follow-up Periods in Patients with MMN

Characteristic, n (%) [95% CI]	Baseline (N = 248)[a]	Follow-up (N = 248)[a]
Specialty submitting the MMN-related index claim		
Primary care	148 (59.7) [53.6-65.8]	NA
Neurologist	14 (5.6) [2.7-8.5]	NA
Other[b]	70 (28.2) [22.6-33.8]	NA
Unknown	16 (6.5) [3.4-9.6]	NA
Diagnostic procedures		
MRI	57 (23.0) [17.8-28.2]	49 (19.8) [14.8-24.8]
Spinal	53 (21.4) [16.3-26.5]	45 (18.1) [13.3-22.9]
Electrophysiological analysis	53 (21.4) [16.3-26.5]	39 (15.7) [11.2-20.2]
Nerve conduction study	49 (19.8) [14.8-24.8]	36 (14.5) [10.1-18.9]
Electromyography	44 (17.7) [12.9-22.5]	38 (15.3) [10.8-19.8]
MMN-associated antibody tests[c]	14 (5.6) [2.7-8.5]	15 (6.0) [3.0-9.0]
Nerve biopsy	NR	NR
Lumbar puncture CSF analysis	NR	NR

Abbreviations: CI, confidence interval; CSF, cerebrospinal fluid; GM1, GM1 gangliosidosis; MMN, multifocal motor neuropathy; MRI, magnetic resonance imaging; NA, not applicable; NR, not reported.
[a]To protect the privacy of individuals, the counts in some rows have been masked. NR indicates values masked due to small counts (<11).
[b]Other specialties included hospitalist (n = 20), pain medicine (n = 12), surgeon (n = 12), and many other specialties (n = 26).
[c]Monoclonal gammopathies/GM1 antibodies.

Table S4. Mean Healthcare Spending During Baseline and Follow-up Periods for Patients with MMN for Overall Population

	Baseline (N = 248)	Follow-up (N = 248)
All-cause healthcare spending in US$		
Total healthcare spending, mean (95% CI)	26 823 (21 370-32 276)	35 460 (28 650-42 270)
Pharmacy spending, mean (95% CI)	7107 (4249-9965)	9290 (5240-13 340)
Medical spending, mean (95% CI)	$19 716 (15 311-24 121)	$26 170 (20 806-31 534)

Abbreviations: CI, confidence interval; MMN, multifocal motor neuropathy.

Table S5. All-Cause HCRU and Healthcare Spending During Baseline and Follow-up Periods for Patients with MMN and No Cancer Diagnosis[a]

	Baseline (N = 177)	Follow-up (N = 177)
**All-cause HCRU**		
Inpatient stays, n (%) [95% CI]	42 (23.7) [17.4-30.0]	47 (26.6) [20.1-33.1]
Total LOS in days, mean (SD) [95% CI]		
All patients	3.1 (9.5) [1.7-4.5]	3.9 (11.0) [2.3-5.5]
Patients with inpatient stays LOS per hospitalization	12.9 (16.0) [8.1-17.7]	14.7 (17.4) [9.7-19.7]
LOS per hospitalization	9.4 (12.9) [5.5-13.3]	9.8 (11.6) [6.5-13.1]
Physician office visit, n (%) [95% CI]	170 (96.0) [93.1-98.9]	174 (98.3) [96.4-100.2]
Outpatient visit,[b] n (%) [95% CI]	166 (93.8) [90.2-97.4]	165 (93.2) [89.5-96.9]
Emergency department visit, n (%) [95% CI]	75 (42.4) [35.1-49.7]	84 (47.5) [40.1-54.9]
Telehealth services, n (%) [95% CI]	29 (16.4) [10.9-21.9]	53 (29.9) [23.2-36.6]
**All-cause healthcare spending in US$**		
Total healthcare spending, median (Q1, Q3)	9345 (3941-21 465)	13 663 (5881-31 594)
Pharmacy spending, median (Q1, Q3)	1290 (512-4851)	1565 (537-5870)
Medical spending, median (Q1, Q3)	6207 (2639-14 130)	9728 (3565-25 332)

Abbreviations: CI, confidence interval; HCRU, healthcare resource utilization; LOS, length of stay; MMN, multifocal motor neuropathy; Q, quartile; SD, standard deviation.
[a]No cancer diagnosis during the baseline or follow-up periods.
[b]Includes all outpatient visits other than physician outpatient appointments and telehealth services.
